# Systematic review of 99 extremity bone malignancy survival prediction models

**DOI:** 10.1186/s10195-025-00821-6

**Published:** 2025-01-28

**Authors:** Cheng-Yo Lai, Hung-Kuan Yen, Hao-Chen Lin, Olivier Quinten Groot, Wei-Hsin Lin, Hao-Ping Hsu

**Affiliations:** 1 Department of Orthopedic Surgery, National Taiwan University Hospital Hsinchu Branch, Hsinchu, Taiwan; 2 Department of Orthopedic Surgery, National Taiwan University Hospital, Taipei, Taiwan; 3 Department of Medical Education, National Taiwan University Hospital, Taipei, Taiwan; 4 Department of Orthopaedics, University Medical Center Utrecht, Utrecht, The Netherlands; 5 Department of Medical Imaging, Chi Mei Medical Center, Tainan, Taiwan

**Keywords:** Extremity bone malignancy, Extremity metastasis, Sarcoma, Prediction models, Systematic review

## Abstract

**Background:**

Various prediction models have been developed for extremity metastasis and sarcoma. This systematic review aims to evaluate extremity metastasis and sarcoma models using the utility prediction model (UPM) evaluation framework.

**Methods:**

We followed Preferred Reporting Items for Systematic Reviews and Meta-Analyses (PRISMA) guidelines and systematically searched PubMed, Embase, and Cochrane to identify articles presenting original prediction models with 1-year survival outcome for extremity metastasis and 5-year survival outcome for sarcoma. Identified models were assessed using the UPM score (0–16), categorized as excellent (12–16), good (7–11), fair (3–6), or poor (0–2). A total of 5 extremity metastasis and 94 sarcoma models met inclusion criteria and were analyzed for design, validation, and performance.

**Results:**

We assessed 5 models for extremity metastasis and 94 models for sarcoma. Only 4 out of 99 (4%) models achieved excellence, 1 from extremity metastasis and 3 from sarcoma. The majority were rated good (62%; 61/99), followed by fair (31%, 31/99) and poor (3%, 3/99).

**Conclusions:**

Most predictive models for extremity metastasis and sarcoma fall short of UPM excellence. Suboptimal study design, limited external validation, and the infrequent availability of web-based calculators are main drawbacks.

**Level of evidence:**

This study is classified as Level 2a evidence according to the Oxford 2011 Levels of Evidence.

*Trial registration* This study was registered in PROSEPRO (CRD42022373391, https://www.crd.york.ac.uk/prospero/display_record.php?RecordID=373391).

**Supplementary Information:**

The online version contains supplementary material available at 10.1186/s10195-025-00821-6.

## Introduction

Extremity bone malignancies encompass a diverse range of cancers affecting the bones of the arms and legs, including metastatic cancers and primary bone sarcomas. Management of bone malignancies requires a multifaceted approach involving systemic treatments such as chemotherapy or targeted therapy, radiation therapy, and surgical interventions [1, 2]. Surgical intervention is commonly necessary when mechanical stability is compromised, as in cases of fracture or impending fracture.

In 2024, an estimated 3970 new cases of bone or joint cancer were diagnosed in the USA, with approximately 2050 deaths [3]. Although rare, these malignancies carry significant morbidity due to their aggressive nature and potential for functional impairment [4, 5]. The selection of an appropriate treatment strategy, particularly surgical intervention, is influenced by an estimation of the patient’s life expectancy [2, 6]. Accurate prognostication is pivotal in tailoring treatments to patients’ needs and optimizing the balance between curative and palliative interventions.

Nevertheless, clinical life expectancy predictions can sometimes be inaccurate, potentially leading to inappropriate or suboptimal treatment decisions [7]. Numerous prediction models have been developed to enhance predictive accuracy for patients with extremity cancer by considering various clinical, pathological, and demographic factors. Many of these models, including those reviewed in this study, represent efforts to address the challenges of prognostication. However, despite their potential, many of these models have yet to undergo external validation or achieve widespread clinical implementation. Given the increasing number of these prediction models, systematically comparing them can provide clinicians with a current overview of the available models, how they perform, and their quality.

The utility of prediction model (UPM) score is an evaluation framework for comparing prediction models’ quality, transparency, and usability [8]. The UPM score considers eight critical prediction model factors, including sample size, institutional diversity, study design, discrimination assessed through the area under the curve (AUC), internal and external validation, model calibration, and the availability of a web-based calculator, which promote the practical usability of these models [9].

In this systematic review, we aimed to evaluate survival prediction models for extremity metastasis and sarcoma using the UPM score framework. Our primary research question focused on systematically comparing the accuracy, applicability, and validation status of these models for extremity bone malignancies. This review includes patients with extremity bone metastasis and sarcoma, assessing each model’s study design, internal and external validation, clinical usability, and predictive outcomes. These evaluations support model comparisons and can inform more practical prognostication and treatment decisions in managing extremity bone cancers.

## Methods

### Literature search

We conducted this systematic review following a protocol registered in PROSEPRO (CRD42022373391). The protocol was guided by Preferred Reporting Items for Systematic Reviews and Meta-Analyses (PRISMA) guidelines [10]. A systematic search was conducted to identify relevant articles on extremity metastasis and sarcoma survival prediction models across three medical databases, PubMed, Embase, and Cochrane (Supplementary Tables I and II). Three reviewers independently screened titles, abstracts, and full-text articles for eligibility, with a senior reviewer resolving any discrepancies.

## Inclusion criteria

The inclusion criteria are modified from the original UPM score article [8]. Articles were eligible if they met the following requirements: (1) developed an original clinical prediction model specifically focused on extremity metastasis or any types of sarcoma; (2) included patients diagnosed with extremity metastasis or any pathology types of sarcoma; (3) reported a survival endpoint with area under the curve (AUC) of 1 year (extremity metastasis) or 5 years (sarcoma); (4) published within the last 20 years (1 January 2003—31 December 2022); and (5) provide full-text content in English online. Articles were excluded if they: (1) were non-peer-reviewed articles or conference abstracts or (2) did not focus on extremity metastasis or sarcoma; and (3) exclusively include patients with non-extremity sarcoma.

The choice of a 1-year survival endpoint for extremity metastasis models was made because metastasis often signifies an advanced and potentially terminal cancer stage. In cases of extremity metastasis, the primary objective of surgical treatment is to alleviate symptoms and improve functionality. When a patient’s life expectancy extends beyond 1 year, the advantages of the surgical procedure could justify the potential perioperative risks and discomfort. The 1-year endpoint was chosen by not only the original UPM article [8], but also other models proposed for decision-making [11–13].

A 5-year survival endpoint was chosen for sarcoma models. Like other primary malignancies, the 5-year survival rate serves as a crucial metric for assessing the aggressiveness of these diseases [14, 15]. This timeframe is widely adopted in sarcoma prediction models for analysis purposes.

### Study selection

**Fig. 1:**
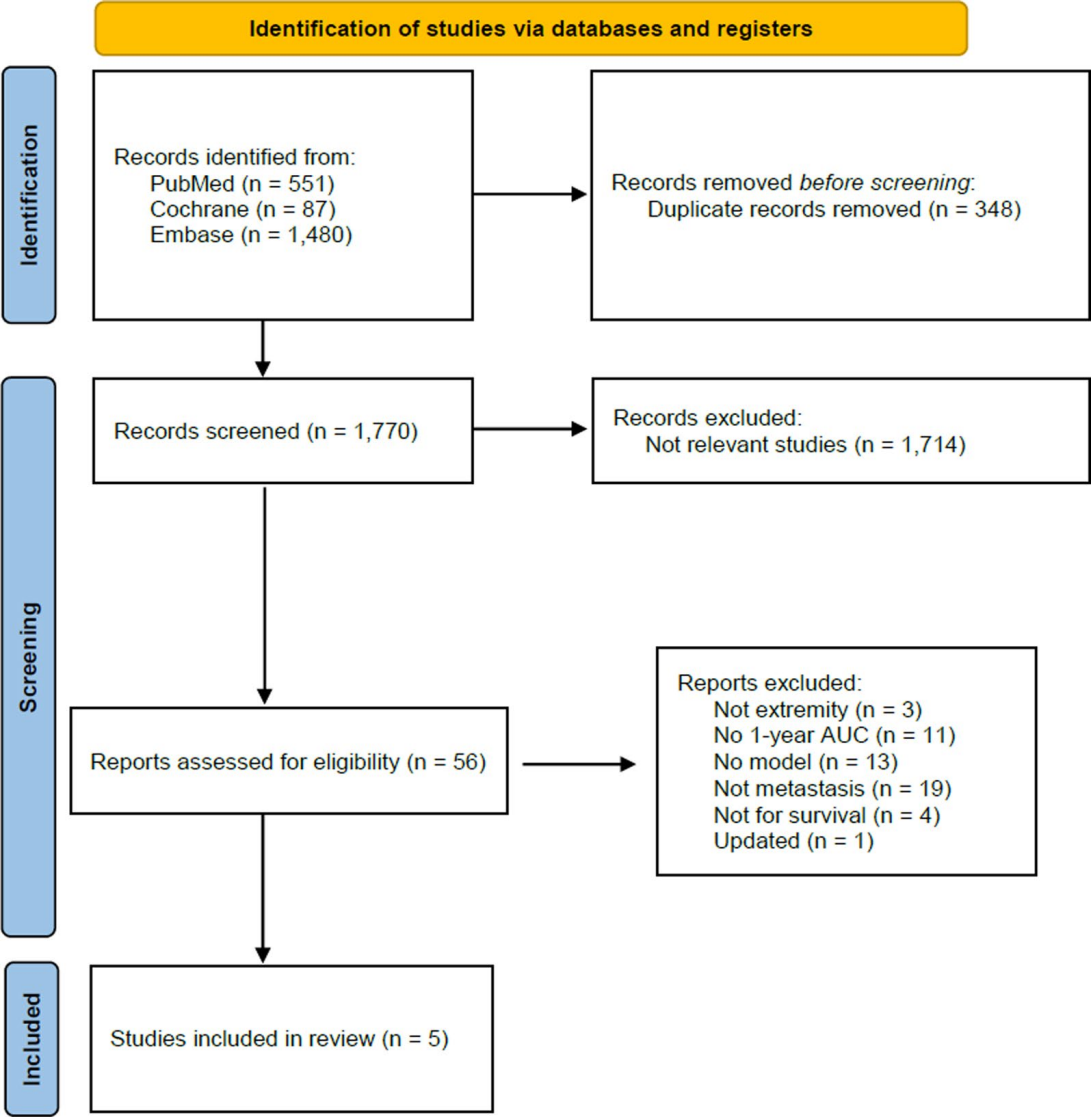
PRISMA flow diagram for extremity metastasis prediction models with 1-year survival AUC

For extremity metastases, 1770 unique articles were identified. After title and abstract screening, 56 full-text articles were assessed, and 5 models were deemed eligible for inclusion in the analysis (Fig. 1).

**Fig. 2:**
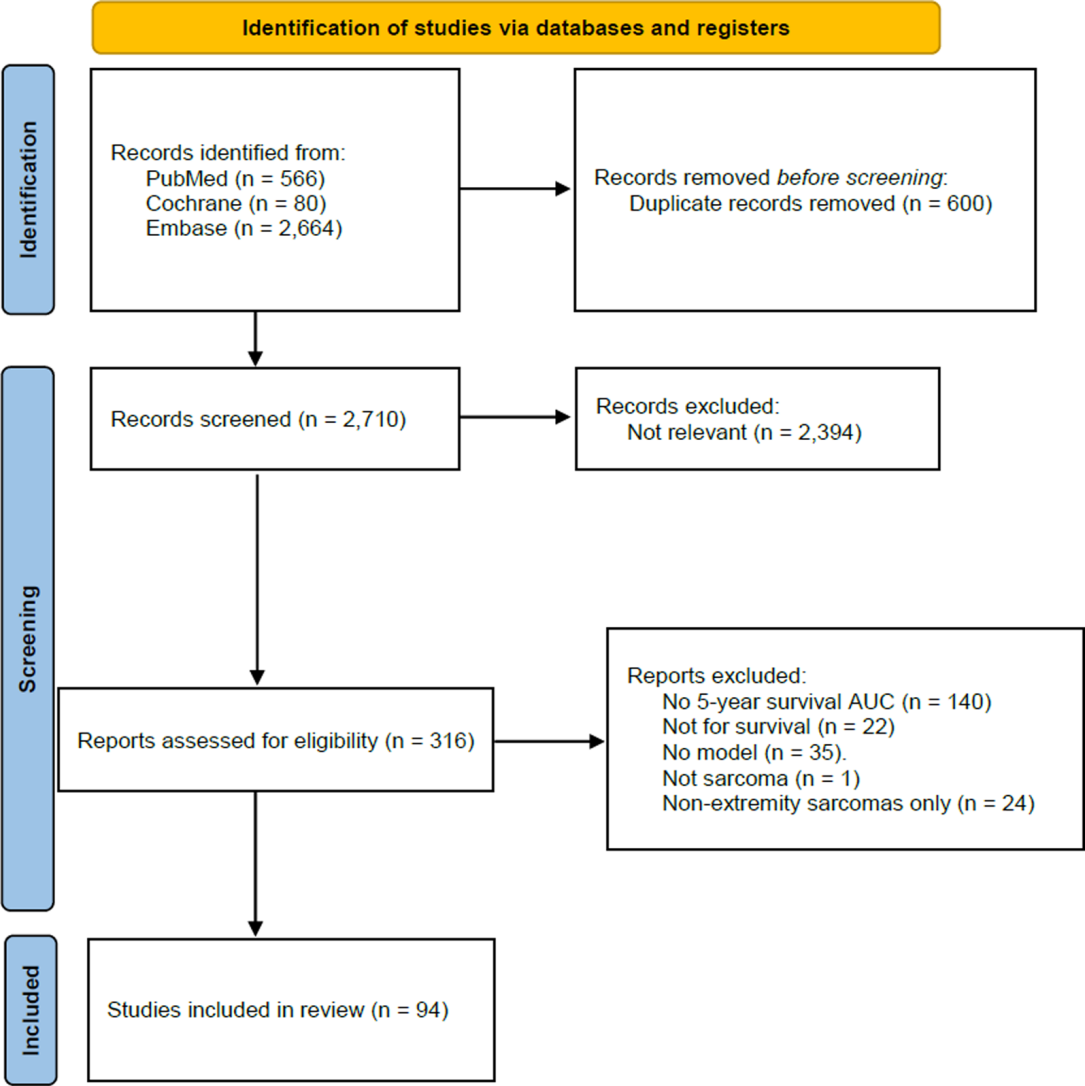
PRISMA flow diagram for sarcoma prediction models with 5-year survival AUC

For sarcoma, 2710 unique articles were identified. Following title and abstract screening, 318 articles were assessed in full text. Finally, 94 models were included in the analyses (Fig. 2).

### UPM score

The UPM score [8] was employed as the evaluation framework, with a score ranging between 0 and 16, and 8 criteria were considered: design, sample size, number of institutions involved, AUC at the specified 1-year or 5-year endpoint, presence of internal validation, weighted AUC from external validations, and availability of a web-based calculator (Table 1). In cases in which an article included multiple survival outcomes (e.g., overall survival, disease-free survival, event-free survival), we prioritized overall survival and adopted the highest AUC value. Some development articles provided validations with AUC, and they were categorized as external validations if they were based on data from different databases or patient cohorts. The weighted external AUC was calculated as the weighted average of AUC, which means the summation of AUC values multiplied by the sample size of the corresponding external validation study and then divided by the total number of patients in all external validation studies. A model’s UPM score was categorized as follows: 0–2 as poor, 3–6 as fair, 7–11 as good, and 12–16 as excellent.

**Table 1:** Utility of prediction model (UPM) scoring system

Model characteristics	Points
Original study sample size	
< 150 patients	0
150–500 patients	1
> 500 patients	2
Original study institution	
Single	0
Binary	1
Multiple	2
Original study design	
Retrospective	0
Combined (retrospective + prospective)	1
Prospective	2
Original study AUC	
< 0.70	0
0.70–0.80	1
> 0.80	2
Internal validation	
No	0
Yes	2
Calibration	
No	0
Yes	2
Weighted external AUC	
< 0.70 or not provided	0
0.70–0.80	2
> 0.80	3
Clinical usability	
No web-based calculator	0
Web-based calculator provided	1
Total UPM scoring	
Poor	0–2
Fair	3–6
Good	7–11
Excellent	12–16

*AUC* area under the curve

### Statistical analysis

The total UPM scores were calculated for each separate model. The completeness of each of the eight categories within the UPM score was presented in percentages and visualized by graph bars. We used Microsoft Excel version 16 to extract and record data using standardized forms by three reviewers and to create figures for result visualization. Endnote version 21 was employed as the reference management system.

## Results

### Extremity metastasis

The 5 models’ UPM scores ranged from 5 to 13 points [11, 16–19]. In addition, 2 models [11, 19] performed calibrations and made their models accessible through web-based calculators and 3 models underwent external validation; 2 models were externally validated by independent studies [11, 19], while the third model conducted external validation using an external database [18] (Table 2).

**Table 2:** Prediction models for 1-year survival of patients with extremity metastasis (*n* = 5)

Study	Ins	Design	Sample	Original AUC	Internal validation	Calibration	External validation	External AUC	WBC	UPMS
Thio et al. [19]	2	Retro	1090	0.81	Yes	Yes	Yes [13, 20]	0.84	Yes	13 (excellent)
Sørensen et al. [11]	1	Combined	270	0.858	No	Yes	Yes [21]	0.802	No	9 (good)
Alcorn et al. [18]	1	Retro	397	0.81	Yes	No	Yes*	0.78	Yes	8 (good)
Janssen et al. [16]	2	Retro	927	0.73	Yes	No	No	-	No	6 (fair)
Errani et al. [17]	1	Pro	159	0.816	No	No	No	-	No	5 (fair)

*Ins* institution, *Retro* retrospective, *Pro* prospective, *AUC* area under the curve, *WBC* web-based calculator, *UPMS* utility of prediction model score

^*^The authors validated the model using different databases or cohorts.

Thio et al. [19] had the highest UPM score of 13. It was a bi-institutional retrospective study with a relatively large sample size of 1090 patients and an AUC of 0.81. The model underwent two external validations with good AUCs by Skalitzky et al. [20] (*n* = 264; AUC 0.83) and Tseng et al. [13] (*n* = 356; AUC 0.84). The model also provided a web-based calculator (https://sorg-apps.shinyapps.io/extremitymetssurvival/).

The model with the next-highest UPM score of 9 was Sørensen et al. [11]. It was a combined study with 270 patients in a single institution. The model achieved a high AUC of 0.87 and was assessed by calibration. Though it had no internal validation, Smolle et al., 2022 [21] (*n* = 303) externally validated it with a high AUC of 0.802.

### Sarcoma

A total of 94 models were included, with 38 published in 2021 and 40 in 2022. Most sarcoma models (90%; 85/94) were developed on the basis of registry or national databases. The Surveillance, Epidemiology, and End Results Program (SEER) was the most chosen training database in 36% (34/94) models, followed by Therapeutically Applicable Research to Generate Effective Treatments (TARGET; 22%; 21/94), The Cancer Genome Atlas (TCGA; 19%; 18/94), and Gene Expression Omnibus (GEO; 13%; 12/94). Most authors contributing to sarcoma prediction models were of Chinese origin. Specifically, of the 95 sarcoma models, 93% (87/94) were developed by Chinese researchers, of which 92% (80/87) used the 4 databases mentioned above. Of these 80 models trained with registry or national databases, 4% (3/80) were externally validated using Chinese patients. [22–24]

Furthermore, 3 models [23–25] achieved excellent UPM scores of 12 or higher (Table 3). All 3 models used retrospective data for training from SEER. They all provided AUCs and calibration, but only one [25] model underwent internal validation. All three models were externally validated; development authors validated two models with a new cohort of hospital patients as the validation group [23, 24], and nondevelopment researchers validated the other [25]. Web-based calculators were provided for all three models (Table 4).

**Table 3:** UPM score distribution of included sarcoma models for 5-year survival prediction (*n* = 99)

Grade	UPMS	Number of models	Total
Poor	0	0	3 (3.0%)
	1	1	
	2	2	
Fair	3	3	30 (30.3%)
	4	8	
	5	4	
	6	15	
Good	7	15	58 (58.6%)
	8	16	
	9	14	
	10	10	
	11	3	
Excellent	12	2	3 (3.0%)
	13	0	
	14	1	
	15	0	
	16	0	

*UPM* utility of prediction model, *UPMS* utility of prediction model score

**Table 4:** Prediction models for 5-year survival of patients with sarcoma with an excellent UPMS (*n* = 6)

Study	Ins	Design	Sample	Original AUC	Internal validation	Calibration	External validation	External AUC	WBC	UPMS
Thio QCBS et al. [25]	Multi	Retro	1544	0.868	Yes	Yes	Yes [26, 27]	0.863	Yes(1)	14
Li W et al. [23]	Multi	Retro	1290	0.855	No	Yes	Yes*	0.834	Yes(2)	12
Li W et al. [24]	Multi	Retro	1144	0.812	No	Yes	Yes*	0.856	Yes(3)	12

*Ins* institution, *Retro* retrospective, *AUC* area under curve, *WBC* web-based calculator, *UPMS* utility of prediction model score

^*^The article authors validated the model using different databases or cohorts

(1) https://sorg-apps.shinyapps.io/chondrosarcoma/

(2) https://drwenle029.shinyapps.io/CHSSapp

(3) https://dr-lee.shinyapps.io/osteosarcoma/

(4) https://lijiayi.shinyapps.io/PGU-RMS_riskcalculator/

Thio et al. [25] had the highest UPM score of 14. It had a large sample size of 1544 patients and achieved an AUC of 0.87. External validation was performed twice by Bongers et al. [26] (*n* = 326; AUC 0.87) using the cohort of two US tertiary care referral centers [27] (*n* = 737; AUC 0.86) as well as the cohort of an Italian tertiary care center. This model also provided a web-based calculator (https://sorg-apps.shinyapps.io/chondrosarcoma/).

### Completeness of UPM score across all models

**Fig. 3:**
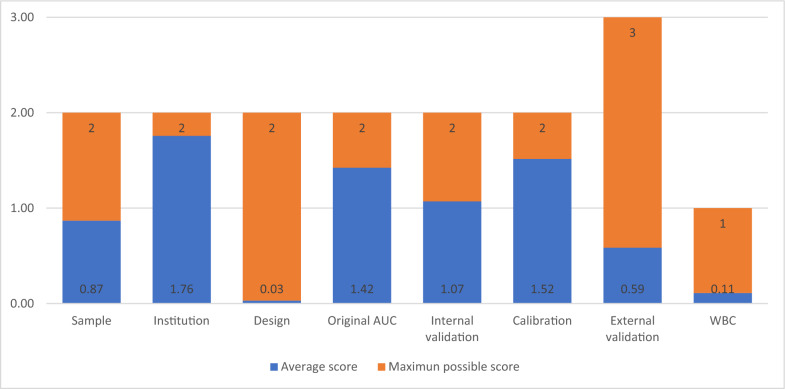
Maximum possible score for each category (blue) of the UPM score framework and the average scores obtained by all 123 models in each category (orange). *AUC* area under curve, *WBC* web-based calculator

The category with the poorest performance was design, where 97/99 (98%) studies received a score of 0 as they were retrospective. The second worst-performing category was the provision of web-based calculators, as 11/99 (11%) provided an online tool. Though 24 out of 99 (24%) models had external validations, most were developed in derivation studies using alternate databases or populations, resulting in 0.59 out of 3 points in the external validation category. Notable, only three models [11, 19, 25] were externally validated by independent researchers. The best-performing category was institution number, with 86% (85/99) of all models being considered multi-institutional, as they were developed on the basis of databases (Fig. 3).

## Discussion

Most prediction models in extremity metastasis and primary sarcomas did not demonstrate excellent performance on the basis of the UPS framework. Only 4 out of 99 models achieved excellence, 1 from extremity metastasis and 3 from sarcoma. The low number of excellent-performing models underscores the need to develop accurate and reliable prediction models for both extremity bone cancers and sarcomas, thereby providing valuable tools for clinical decision-making and patient care.

### Study population

The five included studies of extremity metastasis were constructed on cohorts of patients from one or two hospitals. In contrast, most sarcoma models were developed on the basis of four registry or national databases: SEER, TARGET, TCGA, and GEO. While the SEER, TARGET, and TCGA databases primarily gather patient data from the USA and Canada, it is noteworthy that Chinese researchers conducted a considerable proportion of studies on sarcoma prediction models. However, only a few of them were externally validated with Chinese patients. It is crucial to emphasize the importance of assessing the applicability of prediction models across distinct geographical areas before their widespread use in local patient care. In addition, proposing a universal guideline for sarcoma management in Asian populations is challenging due to the substantial biological and ecosocial diversity across Asia [28, 29]. Consequently, there is a clear need for a more extensive and diverse representation of Asian patient data in developing and validating sarcoma prediction models.

### Poorly performing UPM criteria

On the basis of the observed shortcomings in meeting certain UPM criteria, we propose several considerations. First, a prospective study is typically ranked higher than a retrospective in the hierarchy of study designs [30], as it offers advantages in terms of the accuracy of data collection [31]. Indeed, constructing a prediction model with sufficiently large cohorts is ideal [32] yet challenging, particularly for rare diseases such as sarcoma [33]. Establishing long-term multi-institutional collaborations or databases could be potential solutions [33], although it may come with significant costs and potential compromises in data collection accuracy.

Second, the lack of external validations indicates poor validity of models, and most of these validations were not performed by independent researchers in this systematic review. Previous studies [34, 35] have also shown similar results regarding low quality and quantity of external validations. In addition, a minimum of 100 events in both event and non-event groups is required [36]. It is difficult to conduct an external validation in a single center for rare diseases such as sarcoma, which could explain why most models only conducted external validations on the basis of registry or national databases to meet the required 100 events.

Third, for complex models, the availability of a web-based calculator can facilitate implementation [37, 38]. While such calculators do not directly validate a model, their existence enhances the likelihood of model scrutiny, external validation, and potential adoption in clinical practice. Thus, emphasis should be placed on providing online tools to allow for the evaluation of prediction models with independent data to ensure their generalizability and reproducibility [34, 39–42].

### Compromise in study inclusion

This systematic review was originally designed to analyze exclusively models for extremity malignancies. However, most studies analyzed focused on specific sarcoma pathology types rather than cancer sites and often utilized data from large databases that included both extremity and non-extremity sarcomas. Approximately 43% of sarcoma cases were extremity sarcomas [43], and it is reasonable to infer a similar proportion within the databases used by the included studies. Consequently, the models developed from these mixed populations may still hold relevance and applicability for patients with extremity sarcoma. To maintain this review’s focus, studies exclusively centered on non-extremity sarcomas were excluded. While this approach broadens the scope, it also introduces potential heterogeneity, as non-extremity cases might influence the reported prognostic factors and model performance. This compromise underscores the need for further research specifically targeting extremity sarcomas to enhance the development and validation of tailored prediction models for this subgroup.

### Frequent publication

Interestingly, we observed a discernible trend in the frequent publication of multiple articles by the same authors or institutions in a relatively short timeframe. For example, Li W authored two papers [23, 24] in 2022, both of which received high UPM scores of 12. Such patterns raise the possibility that researchers or institutions consistently utilizing these databases might inadvertently introduce similar biases or overlook specific aspects of their research [44]. This again emphasizes the importance of external validation since models without independent external validation might have misleadingly high performance [34].

### Clinical implementation

In navigating the abundance of available prediction models, especially in the context of sarcoma, several critical steps must be considered. First, accurate models based on high-quality data are essential to ensuring reliability and transparency. Second, validation across distinct geographical areas is imperative to assess the generalizability of these models [28, 29]. Third, validations should ideally be conducted using prospective designs with complete datasets, as this ensures optimal utilization of the models [45, 46]. Fourth, continuous and regular retraining or updating of the models is crucial, considering the evolving landscape of medicine and the potential for data to become outdated over time [46]. Last but not least, prioritizing user friendliness, such as providing simple formulas or web-based calculators, can significantly enhance the clinical practicality of prediction tools [37, 38]. These steps should be undertaken; otherwise, both patients and clinicians cannot take full advantage of the prediction decision tools.

### Limitations

This study and the UPM score framework have several limitations. First, the UPM score was initially designed for spine metastasis or degenerative spine disease. However, we found the included UPM factors pertinent to our context and have adopted the framework for evaluating extremity metastasis and sarcoma. Second, while the UPM score offers a grading system for prediction models, it cannot be applied to all models. For example, the Sarculator [47] and Memorial Sloan Kettering Cancer Center (MKSCC) [48] sarcoma nomograms are commonly used models. Still, this study did not include them because they only provided the Harrell C index rather than the 5-year survival AUC. Third, the UPM score framework may not comprehensively reflect the actual value of models in clinical practice. As mentioned above, some questionable models may have high UPM scores. Fourth, in the UPM score, only the weighted AUC determines the points of external validations. However, the value of effective external validation is not solely determined by performance; factors such as design, sample size, and independence also impact the reliability of external validations [34]. Therefore, future research should aim to develop an effective scoring system for evaluating prediction models and external validations that address these limitations. Fifth, while we focused on survival, other outcomes are as important in treatment decision-making. Although we did not include nonsurvival outcomes, we believe a paucity of studies focus on outcomes such as length of hospital stay, complications, and most importantly, quality of life. Future studies should focus on these evenly important outcomes. Lastly, a limitation of this study is the absence of randomized controlled trials (RCTs) among the included sources, as no eligible RCTs were identified. Since no RCT-based models were available to validate the UPM score framework, this may introduce potential bias in assessing its generalizability and validity.

## Conclusions

Most predictive models for extremity metastasis and sarcoma fall short of UPM excellence. Suboptimal study design, limited external validation, and the infrequent availability of web-based calculators are the main drawbacks. Future work should focus on enhancing model development using a wider range of prospective data and validations, ultimately improving patient care and treatment decisions for extremity bone malignancies.

## Supplementary Information

Supplementary Material 1.

Supplementary Material 2.

Supplementary Material 3.

## Data Availability

The datasets supporting the conclusions of this article are available from the following repositories: Surveillance, Epidemiology, and End Results Program (SEER): Repository: SEER Database; Data used: SEER*Stat Database (1975–2016); Access URL: https://seer.cancer.gov/data/; Note: Access to SEER data requires registration and agreement to data use policies. Therapeutically Applicable Research to Generate Effective Treatments (TARGET): Repository: TARGET Data Matrix; Data used: TARGET Osteosarcoma Dataset; Access URL: https://ocg.cancer.gov/programs/target/data-matrix; Note: Data access may require application and approval through the database of Genotypes and Phenotypes (dbGaP). The Cancer Genome Atlas (TCGA): Repository: TCGA Data Portal; Data used: TCGA Sarcoma (SARC) Project; Access URL: https://portal.gdc.cancer.gov/projects/TCGA-SARC; Note: Data is available under controlled access; users must comply with TCGA data usage policies. Gene Expression Omnibus (GEO): Repository: NCBI GEO Database; Data used: GEO Series GSE123456; Access URL: https://www.ncbi.nlm.nih.gov/geo/query/acc.cgi?acc=GSE123456; Note: Data is publicly available. Extremity Metastasis Survival Prediction Model: Project name: SORG Extremity Metastasis Survival Calculator; Access URL: https://sorg-apps.shinyapps.io/extremitymetssurvival/; License: Open access; Any restrictions: None. Chondrosarcoma Survival Calculator: Project name: SORG Chondrosarcoma Survival Calculator; Access URL: https://sorg-apps.shinyapps.io/chondrosarcoma/; License: Open access. Any restrictions: None. Osteosarcoma Survival Calculator: Project name: Osteosarcoma Survival Prediction Tool; Access URL: https://dr-lee.shinyapps.io/osteosarcoma/; License: Open access; Any restrictions: None. Pediatric Genitourinary Rhabdomyosarcoma Calculator: Project name: PGU-RMS Risk Calculator; Access URL: https://lijiayi.shinyapps.io/PGU-RMS_riskcalculator/; License: Open access; Any restrictions: None. Due to licensing restrictions, processed datasets used during the current study are not publicly available but are available from the corresponding author on reasonable request and with permission from the respective repositories. For further inquiries regarding the datasets and materials, please contact the corresponding author.

## Abbreviations

UPM: Utility prediction model
AUC: Area under the curve
PRISMA: Preferred Reporting Items for Systematic Reviews and Meta-Analyses
SEER: Surveillance, Epidemiology, and End Results program
TARGET: Therapeutically Applicable Research to Generate Effective Treatments
TCGA: The Cancer Genome Atlas
GEO: Gene Expression Omnibus
CI: Confidence interval
HR: Hazard ratio
UPMS: Utility of prediction model score
