# Supplementary material for: Systematic review of 99 extremity bone malignancy survival prediction models
Source: J Orthop Traumatol. 2025 Jan 28;26:5. doi: 10.1186/s10195-025-00821-6 (PMC11775353; doi:10.1186/s10195-025-00821-6)
Supplement: Supplementary file 1 — Supplementary Material 1. [file 10195_2025_821_MOESM1_ESM.docx]

Appendix Table 1. Search syntaxes of extremity metastasis prediction models for the Pubmed, Embase, and Cochrane Databases

| Database | Pubmed | Embase | Cochrane Database |
| --- | --- | --- | --- |
| Date | 2023/01/10 | 2023/01/10 | 2023/01/10 |
| Number of hits | 551 | 87 | 1480 |
| Search strategy | "extremity" OR "extremities" OR "femur" OR "femoral" OR "humerus" OR "humeral" OR "long bone" OR "long-bone" OR "extremities" [Mesh] or "Bones of Upper Extremity" [Mesh] or "Bones of Lower Extremity" [Mesh]) AND ("metastasis" OR "metastases" OR "metastatic" OR "acrometastasis") AND ("prognostic" or "prognosis" OR "survival" OR "mortality") AND ("predict" OR "prediction model" or "nomogram" or "score" OR "scoring system") | ('extremity' OR 'extremities' OR 'femur' OR 'femoral' OR 'humerus' OR 'humeral' OR 'long bone' OR 'long-bone' OR 'limb'/exp or 'bones of extremity'/exp) AND ("metastasis" OR "metastases" OR "metastatic" OR "acrometastasis") AND ("prognostic" or "prognosis" OR "survival" OR "mortality") AND ("predict" OR "prediction model" or "nomogram" or "score" OR "scoring system") | "extremity" OR "extremities" OR "femur" OR "femoral" OR "humerus" OR "humeral" OR "long bone" OR "long-bone" OR "extremities" [Mesh] or "Bones of Upper Extremity" [Mesh] or "Bones of Lower Extremity" [Mesh]) AND ("metastasis" OR "metastases" OR "metastatic" OR "acrometastasis") AND ("prognostic" or "prognosis" OR "survival" OR "mortality") AND ("predict" OR "prediction model" or "nomogram" or "score" OR "scoring system") |

Appendix Table 2. Search Syntaxes of sarcoma prediction models for the Pubmed, Embase, and Cochrane Databases

| Database | Pubmed | Embase | Cochrane Database |
| --- | --- | --- | --- |
| Date | 2023/02/05 | 2023/02/05 | 2023/02/05 |
| Number of hits | 566 | 80 | 2664 |
| Search strategy | ("sarcoma") AND ("prognostic" or "prognosis" OR "survival" OR "mortality") AND ("predict" OR "prediction model" or "nomogram" or "prediction score" OR "predict score" OR "scoring system") | ("sarcoma") AND ("prognostic" or "prognosis" OR "survival" OR "mortality") AND ("predict" OR "prediction model" or "nomogram" or "prediction score" OR "predict score" OR "scoring system") | ("sarcoma") AND ("prognostic" or "prognosis" OR "survival" OR "mortality") AND ("predict" OR "prediction model" or "nomogram" or "prediction score" OR "predict score" OR "scoring system") |
