# Supplementary material for: Systematic review of 99 extremity bone malignancy survival prediction models
Source: J Orthop Traumatol. 2025 Jan 28;26:5. doi: 10.1186/s10195-025-00821-6 (PMC11775353; doi:10.1186/s10195-025-00821-6)
Supplement: Supplementary file 2 — Supplementary Material 2. [file 10195_2025_821_MOESM2_ESM.docx]

| **Supplementary table 1.** Evaluation of Sarcoma models using the UPMS (in alphabetical order of study title) | | | | | | | | | | |  |
| --- | --- | --- | --- | --- | --- | --- | --- | --- | --- | --- | --- |
| **Study** | **Institutional number or used database** | **Design** | **Sample** | **Original AUC** | **Internal validation** | **Calibration** | **External validation** | **External AUC** | **Web-based calculator** | **UPMS** |  |
| [1] | TARGET | Retro | 84 | 0.881 | No | No | Yes* | 0.849 | No | 7 |  |
| [2] | SEER | Retro | 1583 | 0.812 | Yes | Yes | No |  | No | 10 |  |
| [3] | TCGA | Retro | 640 | 0.729 | No | Yes | No |  | No | 7 |  |
| [4] | SEER | Retro | 1544 | 0.868 | Yes | Yes | Yes_­_^†^ | 0.86 | Yes | 14 |  |
| [5] | GEO | Retro | 103 | 0.943 | No | Yes | Yes* | 0.81 | No | 9 |  |
| [6] | SEER | Retro | 393 | 0.766 | Yes | Yes | No |  | No | 8 |  |
| [7] | TARGET | Retro | 88 | 0.84 | No | Yes | Yes* | 0.73 | No | 8 |  |
| [8] | GEO | Retro | 53 | 0.71 | No | Yes | Yes* | 0.73 | No | 7 |  |
| [9] | TARGET | Retro | 88 | 0.771 | No | Yes | Yes* | 0.744 | No | 7 |  |
| [10] | TARGET | Retro | 93 | 0.865 | No | No | Yes* | 0.618 | No | 4 |  |
| [11] | TARGET | Retro | 85 | 0.938 | Yes | Yes | No |  | No | 8 |  |
| [12] | GEO | Retro | 41 | 0.813 | No | Yes | Yes* | 0.724 | No | 8 |  |
| [13] | GEO | Retro | 88 | 0.90 | Yes | Yes | No |  | No | 8 |  |
| [14] | TCGA | Retro | 85 | 0.797 | Yes | No | No |  | No | 5 |  |
| [15] | TARGET | Retro | 85 | 0.85 | Yes | No | Yes* | 0.65 | No | 6 |  |
| [16] | SEER | Retro | 1094 | 0.793 | No | Yes | Yes* | 0.814 | Yes | 11 |  |
| [17] | 3 | Retro | 242 | 0.814 | Yes | No | No |  | No | 7 |  |
| [18] | SEER | Retro | 3145 | 0.895 | Yes | Yes | No |  | Yes | 11 |  |
| [19] | TARGET | Retro | 93 | 0.92 | Yes | Yes | No |  | No | 8 |  |
| [20] | TARGET | Retro | 97 | 0.66 | No | Yes | Yes* | 0.61 | No | 4 |  |
| [21] | SEER | Retro | 1687 | 0.811 | Yes | Yes | No |  | No | 10 |  |
| [22] | SEER | Retro | 4505 | 0.752 | Yes | Yes | No |  | No | 9 |  |
| [23] | SEER | Retro | 903 | 0.816 | Yes | Yes | No |  | No | 10 |  |
| [24] | SEER | Retro | 5733 | 0.828 | Yes | Yes | No |  | No | 10 |  |
| [25] | 1 | Retro | 454 | 0.71 | Yes | No | No |  | No | 4 |  |
| [26] | SEER | Retro | 1270 | 0.752 | Yes | Yes | No |  | No | 9 |  |
| [27] | SEER | Retro | 2114 | 0.765 | Yes | Yes | No |  | Yes | 10 |  |
| [28] | TARGET | Retro | 88 | 0.889 | No | Yes | Yes* | 0.672 | No | 6 |  |
| [29] | 1 | Retro | 198 | 0.80 | No | Yes | Yes* | 0.78 | No | 6 |  |
| [30] | SEER | Retro | 846 | 0.80 | Yes | Yes | No |  | No | 9 |  |
| [31] | TCGA | Retro | 259 | 0.738 | Yes | Yes | No |  | No | 8 |  |
| [32] | TCGA | Retro | 255 | 0.834 | No | Yes | No |  | No | 7 |  |
| [33] | TCGA | Retro | 80 | 0.968 | Yes | No | No |  | No | 6 |  |
| [34] | SEER | Retro | 1290 | 0.855 | No | Yes | Yes* | 0.834 | Yes | 12 |  |
| [35] | SEER | Retro | 776 | 0.764 | Yes | Yes | No |  | No | 9 |  |
| [36] | SEER | Retro | 11424 | 0.81 | Yes | Yes | No |  | No | 10 |  |
| [37] | TCGA | Retro | 263 | 0.699 | No | Yes | Yes* | 0.609 | No | 5 |  |
| [38] | TARGET | Retro | 85 | 0.749 | No | Yes | Yes* | 0.717 | No | 7 |  |
| [39] | GEO | Retro | 53 | 0.8 | No | Yes | Yes* | 0.7 | No | 7 |  |
| [40] | TARGET | Retro | 93 | 0.838 | No | Yes | Yes* | 0.651 | No | 6 |  |
| [41] | TARGET | Retro | 82 | 0.85 | No | Yes | Yes* | 0.85 | No | 7 |  |
| [42] | 1 | Retro | 223 | 0.80 | Yes | Yes | No |  | No | 8 |  |
| [43] | TCGA | Retro | 259 | 0.763 | Yes | No | No |  | No | 6 |  |
| [44] | TCGA | Retro | 86 | 0.701 | Yes | Yes | Yes* | 0.757 | No | 9 |  |
| [45] | TARGET | Retro | 88 | 0.883 | No | No | Yes* | 0.771 | No | 6 |  |
| [46] | GEO | Retro | 155 | 0.817 | Yes | No | No |  | No | 7 |  |
| [47] | TARGET | Retro | 85 | 0.907 | No | Yes | No |  | No | 6 |  |
| [48] | TARGET | Retro | 84 | 0.82 | No | No | No |  | No | 4 |  |
| [49] | TCGA | Retro | 260 | 0.596 | No | Yes | No |  | No | 5 |  |
| [50] | TCGA | Retro | 259 | 0.713 | No | Yes | Yes* | 0.735 | No | 8 |  |
| [51] | TARGET+GEO | Retro | 86 | 0.65 | No | No | Yes* | 0.63 | No | 2 |  |
| [52] | TCGA | Retro | 259 | 0.745 | No | Yes | No |  | No | 6 |  |
| [53] | TARGET | Retro | 87 | 0.895 | No | No | No |  | No | 4 |  |
| [54] | TCGA | Retro | 255 | 0.695 | Yes | Yes | No |  | No | 7 |  |
| [55] | TCGA | Retro | 71 | 0.92 | Yes | No | No |  | No | 6 |  |
| [56] | TARGET | Retro | 95 | 0.96 | No | Yes | No |  | No | 6 |  |
| [57] | GEO | Retro | 106 | 0.68 | No | No | Yes* | 0.68 | No | 2 |  |
| [58] | SEER | Retro | 694 | 0.801 | Yes | Yes | No |  | No | 10 |  |
| [59] | SEER | Retro | 1144 | 0.812 | No | Yes | Yes* | 0.856 | Yes | 12 |  |
| [60] | TARGET | Retro | 98 | 0.891 | No | No | No |  | No | 4 |  |
| [61] | GEO | Retro | 53 | 0.752 | No | No | No |  | No | 3 |  |
| [62] | 1 | Retro | 59 | 0.759 | No | No | No |  | No | 1 |  |
| [63] | SEER | Retro | 2643 | 0.718 | Yes | Yes | No |  | No | 9 |  |
| [64] | SEER | Retro | 2009 | 0.805 | Yes | Yes | No |  | No | 10 |  |
| [65] | SEER | Retro | 2059 | 0.712 | Yes | Yes | No |  | No | 9 |  |
| [66] | SEER | Retro | 2170 | 0.839 | Yes | Yes | No |  | No | 10 |  |
| [67] | SEER | Retro | 227 | 0.836 | Yes | Yes | No |  | No | 9 |  |
| [68] | GEO | Retro | 88 | 0.74 | Yes | Yes | Yes* | 0.62 | No | 7 |  |
| [69] | TCGA | Retro | 189 | 0.675 | Yes | Yes | Yes* | 0.621 | No | 7 |  |
| [70] | GEO | Retro | 53 | 0.92 | No | Yes | Yes* | 0.76 | No | 8 |  |
| [71] | TCGA | Retro | 256 | 0.75 | No | Yes | Yes* | 0.64 | No | 6 |  |
| [72] | TCGA | Retro | 259 | 0.681 | Yes | Yes | No |  | No | 7 |  |
| [73] | SEER | Retro | 935 | 0.74 | Yes | Yes | No |  | No | 9 |  |
| [74] | 1 | Retro | 116 | 0.802 | No | Yes | No |  | No | 4 |  |
| [75] | TARGET | Retro | 95 | 0.879 | No | Yes | Yes* | 0.68 | No | 6 |  |
| [76] | SEER | Retro | 346 | 0.822 | Yes | Yes | No |  | No | 9 |  |
| [77] | TCGA | Retro | 86 | 0.906 | No | Yes | Yes* | 0.951 | No | 9 |  |
| [78] | SEER | Retro | 762 | 0.68 | Yes | Yes | No |  | Yes | 9 |  |
| [79] | TCGA | Retro | 85 | 0.731 | No | Yes | No |  | No | 5 |  |
| [80] | SEER | Retro | 835 | 0.911 | Yes | No | No |  | No | 8 |  |
| [81] | SEER | Retro | 357 | 0.779 | Yes | Yes | No |  | No | 8 |  |
| [82] | 1 | Retro | 123 | 0.795 | No | Yes | No |  | No | 3 |  |
| [83] | SEER | Retro | 456 | 0.771 | Yes | Yes | No |  | No | 8 |  |
| [84] | SEER | Retro | 251 | 0.772 | Yes | Yes | No |  | No | 8 |  |
| [85] | GEO | Retro | 53 | 1 | No | No | No |  | No | 4 |  |
| [86] | 1 | Retro | 133 | 0.773 | No | Yes | No |  | No | 3 |  |
| [87] | SEER | Retro | 498 | 0.761 | Yes | Yes | No |  | No | 8 |  |
| [88] | TARGET | Retro | 86 | 0.822 | No | Yes | Yes* | 0.772 | No | 8 |  |
| [89] | SEER | Retro | 1396 | 0.763 | Yes | Yes | No |  | Yes | 10 |  |
| [90] | SEER | Retro | 439 | 0.766 | Yes | Yes | No |  | No | 8 |  |
| [91] | SEER | Retro | 1120 | 0.756 | Yes | Yes | No |  | No | 9 |  |
| [92] | GEO | Retro | 106 | 0.927 | No | Yes | Yes* | 0.694 | No | 6 |  |
| [93] | 1 | Retro | 150 | 0.84 | Yes | Yes | No |  | No | 7 |  |
| [94] | SEER | Retro | 2332 | 0.91 | Yes | Yes | No |  | Yes | 11 |  |
| Retro = retrospective; AUC = area under curve; UPMS = Utility of Prediction Model Score  SEER = The Surveillance, Epidemiology, and End Results Program  TARGET = Therapeutically Applicable Research to Generate Effective Treatments  TCGA = The Cancer Genome Atlas  GEO = Gene Expression Omnibus  *The article author validated the model using different database or cohort.  †This article was externally validated by [95, 96]  [1] D. Zheng, K. Yang, X. Chen, Y. Li, Y. Chen, Analysis of Immune-Stromal Score-Based Gene Signature and Molecular Subtypes in Osteosarcoma: Implications for Prognosis and Tumor Immune Microenvironment, Front Genet (1664-8021 (Print)) (2021).  [2] Z. Wang, J. Liu, J. Han, Z. Yang, Q. Wang, Analysis of prognostic factors of undifferentiated pleomorphic sarcoma and construction and validation of a prediction nomogram based on SEER database, Med Res (2047-783X (Electronic)) (2022).  [3] B. Tu, Y. Jia, J. Qian, Bioinformatics Analysis Identified Five Widely Expressed Genes Associated with Prognosis in Sarcoma, Int J Gen Med (1178-7074 (Print)) (2022).  [4] Q. Thio, A.V. Karhade, P.T. Ogink, K.A. Raskin, K. De Amorim Bernstein, S.A. Lozano Calderon, J.H. Schwab, Can Machine-learning Techniques Be Used for 5-year Survival Prediction of Patients With Chondrosarcoma?, Clin Orthop Relat Res 476(10) (2018) 2040-2048.  [5] G. Man, A.A.-O. Duan, W. Liu, J. Cheng, Y. Liu, J. Song, H. Zhou, K. Shen, Circular RNA-Related CeRNA Network and Prognostic Signature for Patients with Osteosarcoma, Cancer Manag Res (1179-1322 (Print)) (2021).  [6] C.A.-O. Huang, Q.A.-O. Yu, Z.A.-O. Ding, Z.A.-O. Zhou, X.A.-O. Shi, The clinical characteristics, novel predictive tool, and risk classification system for primary Ewing sarcoma patients that underwent chemotherapy: A large population-based retrospective cohort study, Cancer Med (2045-7634 (Electronic)) (2023).  [7] K.A.-O. Xiao, Z.A.-O. Liu, Z.A.-O. Zeng, F.A.-O. Yan, L.A.-O. Xiao, J.A.-O. Li, L.A.-O. Cai, Construction and Validation of a Macrophage-Associated Risk Model for Predicting the Prognosis of Osteosarcoma, J Oncol (1687-8450 (Print)) (2021).  [8] Y. Fu, Z. Jin, Y. Shen, Z. Zhang, M. Li, Z. Liu, G. He, J. Wu, J. Wen, Q. Bao, J. Wang, W. Zhang, Construction and validation of a novel apoptosis-associated prognostic signature related to osteosarcoma metastasis and immune infiltration, Transl Oncol (1936-5233 (Print)) (2022).  [9] S.A.-O. Liu, B.A.-O. Wu, X.A.-O. Li, L.A.-O. Zhao, W.A.-O. Wu, S.A.-O. Ai, Construction and Validation of a Potent Epigenetic Modification-Related Prognostic Signature for Osteosarcoma Patients, J Oncol (1687-8450 (Print)) (2021).  [10] B. Ning, Y. Liu, T. Xu, Y. Li, D. Wei, T. Huang, Y. Wei, Construction and validation of a prognostic model for osteosarcoma patients based on autophagy-related genes, Discov Oncol (2730-6011 (Electronic)) (2022).  [11] S. Ni, J. Hong, W. Li, M. Ye, J.A.-O. Li, Construction of a cuproptosis-related lncRNA signature for predicting prognosis and immune landscape in osteosarcoma patients, Cancer Med (2045-7634 (Electronic)) (2022).  [12] Z. Ouyang, G. Li, H. Zhu, J. Wang, T. Qi, Q. Qu, C. Tu, J. Qu, Q. Lu, Construction of a Five-Super-Enhancer-Associated-Genes Prognostic Model for Osteosarcoma Patients, Front Cell Dev Biol (2296-634X (Print)) (2020).  [13] R.A.-O. Zhao, C.A.-O. Xiong, C.A.-O. Zhang, L.A.-O. Wang, H.A.-O. Liang, X.A.-O. Luo, Construction of a Prognosis-Related Gene Signature by Weighted Gene Coexpression Network Analysis in Ewing Sarcoma, Comput Math Methods Med (1748-6718 (Electronic)) (2022).  [14] Y. He, H. Zhou, H. Xu, H. You, H. Cheng, Construction of an Immune-Related lncRNA Signature That Predicts Prognosis and Immune Microenvironment in Osteosarcoma Patients, Front Oncol (2234-943X (Print)) (2022).  [15] C.A.-O. Dong, Y. Sun, Y. Zhang, B. Qin, T. Lei, Construction of Molecular Subtype and Prognosis Prediction Model of Osteosarcoma Based on Aging-Related Genes, J Oncol (1687-8450 (Print)) (2022).  [16] W. Li, Y. Dong, W. Liu, Z. Tang, C. Sun, S. Lowe, S. Chen, R. Bentley, Q. Zhou, C. Xu, W. Li, B. Wang, H. Wang, S. Dong, Z. Hu, Q. Liu, X. Cai, X. Feng, W. Zhao, C. Yin, A deep belief network-based clinical decision system for patients with osteosarcoma, Front Immunol (1664-3224 (Electronic)) (2022).  [17] I. Han, J.H. Kim, H. Park, H.S. Kim, S.A.-O. Seo, Deep learning approach for survival prediction for patients with synovial sarcoma, Tumour Biol. (1423-0380 (Electronic)) (2018).  [18] L. Yan, N. Gao, F. Ai, Y. Zhao, Y. Kang, J. Chen, Y. Weng, Deep learning models for predicting the survival of patients with chondrosarcoma based on a surveillance, epidemiology, and end results analysis, Front Oncol (2234-943X (Print)) (2022).  [19] X. Wang, C. Xie, L. Lin, Development and validation of a cuproptosis-related lncRNA model correlated to the cancer-associated fibroblasts enable the prediction prognosis of patients with osteosarcoma, J Bone Oncol (2212-1366 (Print)) (2022).  [20] Y. Fu, Q. Bao, Z. Liu, G. He, J. Wen, Q. Liu, Y. Xu, Z. Jin, W. Zhang, Development and Validation of a Hypoxia-Associated Prognostic Signature Related to Osteosarcoma Metastasis and Immune Infiltration, Front Cell Dev Biol (2296-634X (Print)) (2021).  [21] J. Zhang, Z. Pan, F. Zhao, X. Feng, Y. Huang, C. Hu, Y. Li, J.A.-O. Lyu, Development and validation of a nomogram containing the prognostic determinants of chondrosarcoma based on the Surveillance, Epidemiology, and End Results database, Int J Clin Oncol (1437-7772 (Electronic)) (2019).  [22] J. Zhang, J. Yang, H.Q. Wang, Z. Pan, X. Yan, C. Hu, Y. Li, J. Lyu, Development and validation of a nomogram for osteosarcoma-specific survival: A population-based study, Medicine (Baltimore) (1536-5964 (Electronic)) (2019).  [23] J. Zhao, Y. Jiao J Fau - Su, L. Su Y Fau - Mu, L. Mu, Development and validation of a nomogram for specific survival in osteosarcoma patients less than 60 years old: a population-based study, J BUON (2241-6293 (Electronic)) (2021).  [24] S. Cao, J. Li, K. Yang, J. Zhang, J. Xu, C. Feng, H. Li, Development and validation of a novel prognostic model for long-term overall survival in liposarcoma patients: a population-based study, J Int Med Res 48(12) (2020) 300060520975882.  [25] B. Wang, J. Tu, J. Yin, C. Zou, J. Wang, G. Huang, X. Xie, J. Shen, Development and validation of a pretreatment prognostic index to predict death and lung metastases in extremity osteosarcoma, Oncotarget 6(35) (2015) 38348-59.  [26] S. Cao, J. Li, J. Zhang, H. Li, Development and validation of a prognostic nomogram for predicting the overall survival of myxofibrosarcoma patients: a large population-based study, Transl Cancer Res (2219-6803 (Electronic)) (2021).  [27] L. Feng, Y. Chen, T. Ye, Z. Shao, C. Ye, J. Chen, Development and validation of an online prognostic nomogram for osteosarcoma after surgery: a retrospective study based on the SEER database and external validation with single-center data, Cancer Res (2219-6803 (Electronic)) (2022).  [28] J. Hong, Q. Li, X. Wang, J. Li, W. Ding, H. Hu, L.A.-O. He, Development and validation of apoptosis-related signature and molecular subtype to improve prognosis prediction in osteosarcoma patients, J Clin Lab Anal (1098-2825 (Electronic)) (2022).  [29] D. Cheng, X. Qiu, M. Zhuang, C. Zhu, H. Zou, A. Zhang, Development and validation of nomogram based on miR-203 and clinicopathological characteristics predicting survival after neoadjuvant chemotherapy and surgery for patients with non-metastatic osteosarcoma, Oncotarget (1949-2553 (Electronic)) (2017).  [30] X. Liu, S. He, X. Yao, T. Hu, Development and Validation of Prognostic Nomograms for Elderly Patients with Osteosarcoma, Int J Gen Med (1178-7074 (Print)) (2021).  [31] D. Shi, S. Mu, F. Pu, B. Zhong, B. Hu, J. Liu, T. He, Z. Zhang, Z. Shao, Development of a Novel Immune Infiltration-Related ceRNA Network and Prognostic Model for Sarcoma, Front Cell Dev Biol (2296-634X (Print)) (2021).  [32] H. Li, D. Lin, X. Wang, Z. Feng, J. Zhang, K. Wang, The development of a novel signature based on the m(6)A RNA methylation regulator-related ceRNA network to predict prognosis and therapy response in sarcomas, Front Genet (1664-8021 (Print)) (2022).  [33] J. Shi, D. Huang, G. Zhang, F. Zhao, L. Yang, A DNA methylation-associated nomogram predicts the overall survival of osteosarcoma, Medicine (Baltimore) (1536-5964 (Electronic)) (2020).  [34] W. Li, G. Wang, R. Wu, S. Dong, H. Wang, C. Xu, B. Wang, W. Li, Z. Hu, Q. Chen, C. Yin, Dynamic Predictive Models With Visualized Machine Learning for Assessing Chondrosarcoma Overall Survival, Front Oncol 12 (2022) 880305.  [35] K.N. Dai, A.B. Li, An Efficient Nomogram to Predict Overall Survival of Patients with Pediatric Ewing's Sarcoma: A Population-Based Study, Int J Gen Med (1178-7074 (Print)) (2021).  [36] M. Chen, X. He, Q. Yang, J. Zhang, J. Peng, D. Wang, K. Tong, W. Huang, Epidemiology and prediction model of patients with carcinosarcoma in the United States, Front Public Health (2296-2565 (Electronic)) (2022).  [37] Y. Liu, C. Liu, H. Zhang, X. Yi, A. Yu, Establishment of A Nomogram for Predicting the Prognosis of Soft Tissue Sarcoma Based on Seven Glycolysis-Related Gene Risk Score, Front Genet (1664-8021 (Print)) (2021).  [38] J.A.-O. Li, X. Tang, Y. Du, J. Dong, Z. Zhao, H. Hu, T. Song, J. Guo, Y.A.-O. Wang, T. Xu, C. Shao, Y. Sheng, Y.A.-O. Xi, Establishment of an Autophagy-Related Clinical Prognosis Model for Predicting the Overall Survival of Osteosarcoma, Biomed Res Int (2314-6141 (Electronic)) (2021).  [39] Y. Fu, G. He, Z. Liu, J. Wang, Z. Zhang, Q. Bao, J. Wen, Z. Jin, W. Zhang, Exploration and Validation of a Novel Inflammatory Response-Associated Gene Signature to Predict Osteosarcoma Prognosis and Immune Infiltration, J Inflamm Res (1178-7031 (Print)) (2021).  [40] T. Lei, H. Qian, P. Lei, Y.A.-O. Hu, Ferroptosis-related gene signature associates with immunity and predicts prognosis accurately in patients with osteosarcoma, Cancer Sci (1349-7006 (Electronic)) (2021).  [41] Y. Deng, W. Yuan, E. Ren, Z. Wu, G. Zhang, Q. Xie, A four-methylated LncRNA signature predicts survival of osteosarcoma patients based on machine learning, Genomics 113(1, Part 2) (2021) 785-794.  [42] L. Li, Y. Wang, X. He, Z. Li, M. Lu, T. Gong, Q. Chang, J. Lin, C. Liu, Y. Luo, L. Min, Y. Zhou, C. Tu, Hematological Prognostic Scoring System Can Predict Overall Survival and Can Indicate Response to Immunotherapy in Patients With Osteosarcoma, Front Immunol (1664-3224 (Electronic)) (2022).  [43] Z. Lin, Y. Xu, X. Zhang, J. Wan, T. Zheng, H. Chen, S. Chen, T. Liu, Identification and Validation of Pyroptosis-Related lncRNA Signature and Its Correlation with Immune Landscape in Soft Tissue Sarcomas, Int J Gen Med (1178-7074 (Print)) (2021).  [44] D. Zheng, K. Xia, Z. Wei, Z. Wei, W. Guo, Identification of a novel gene signature with regard to ferroptosis, prognosis prediction, and immune microenvironment in osteosarcoma, Front Genet (1664-8021 (Print)) (2022).  [45] L. Fan, J. Ru, T. Liu, C. Ma, Identification of a Novel Prognostic Gene Signature From the Immune Cell Infiltration Landscape of Osteosarcoma, Front Cell Dev Biol (2296-634X (Print)) (2021).  [46] Y.A.-O. Chen, H. Su, Y. Su, Y. Zhang, Y. Lin, F. Haglund, Identification of an RNA-Binding-Protein-Based Prognostic Model for Ewing Sarcoma, Cancers (Basel) (2072-6694 (Print)) (2021).  [47] B. Liu, Z. Liu, C. Feng, C. Li, H. Zhang, Z. Li, C. Tu, S. He, Identification of cuproptosis-related lncRNA prognostic signature for osteosarcoma, Front Endocrinol (Lausanne) (1664-2392 (Print)) (2022).  [48] J. Huang, J. Zhang, H. Xiao, Identification of Epigenetic-Dysregulated lncRNAs Signature in Osteosarcoma by Multi-Omics Data Analysis, Front Med (Lausanne) (2296-858X (Print)) (2022).  [49] Z. Guan, S. Liu, L. Luo, Z. Wu, S. Lu, Z. Guan, K. Tao, Identification of Ferroptosis-Related Genes as Biomarkers for Sarcoma, Front Cell Dev Biol (2296-634X (Print)) (2022).  [50] J. Li, C. Hu, Y. Du, X. Tang, C. Shao, T. Xu, Z. Zhao, H. Hu, Y. Sheng, J. Guo, Y. Xi, Identification of Iron Metabolism-Related Gene Signatures for Predicting the Prognosis of Patients With Sarcomas, Front Oncol (2234-943X (Print)) (2021).  [51] Z. Chen, H. Kong, Z. Cai, K. Chen, B. Wu, H. Li, P. Wang, Y. Wu, H. Shen, Identification of MAP3K15 as a potential prognostic biomarker and correlation with immune infiltrates in osteosarcoma, Ann Transl Med (2305-5839 (Print)) (2021).  [52] W.A.-O. Dai, B. Wang, J.A.-O. Li, Z.A.-O. Luo, Identification of Prognostic lncRNA Related to the Immune Microenvironment of Soft Tissue Sarcoma, Biomed Res Int (2314-6141 (Electronic)) (2022).  [53] W. Huang, Y. Xiao, H. Wang, G. Chen, K. Li, Identification of risk model based on glycolysis-related genes in the metastasis of osteosarcoma, Front Endocrinol (Lausanne) (1664-2392 (Print)) (2022).  [54] D. Dai, L. Xie, Y. Shui, J. Li, Q. Wei, Identification of Tumor Microenvironment-Related Prognostic Genes in Sarcoma, Front Genet (1664-8021 (Print)) (2021).  [55] X. Zhang, Y. Zheng, G. Li, C. Yu, T. Ji, S. Miao, Identifying four DNA methylation gene sites signature for predicting prognosis of osteosarcoma, Transl Cancer Res (2219-6803 (Electronic)) (2020).  [56] Q. Huang, Y. Lin, C. Chen, J. Lou, T. Ren, Y. Huang, H. Zhang, Y. Yu, Y. Guo, W. Wang, B. Wang, J. Niu, J. Xu, L. Guo, W. Guo, Immune-Related LncRNAs Affect the Prognosis of Osteosarcoma, Which Are Related to the Tumor Immune Microenvironment, Front Cell Dev Biol (2296-634X (Print)) (2021).  [57] E.H. Ren, Y.J. Deng, W.H. Yuan, G.Z. Zhang, Z.L. Wu, C.Y. Li, Q.Q. Xie, An Immune-Related Long Non-Coding RNA Signature to Predict the Prognosis of Ewing's Sarcoma Based on a Machine Learning Iterative Lasso Regression, Front Cell Dev Biol (2296-634X (Print)) (2021).  [58] Z. Xing, X. Zhu, Z. Li, H. Wang, M. Qian, X. Zhai, Incidence, clinical characteristics, and prognostic nomograms for patients with myeloid sarcoma: A SEER-based study, Front Oncol (2234-943X (Print)) (2022).  [59] W. Li, G. Jin, H. Wu, R. Wu, C. Xu, B. Wang, Q. Liu, Z. Hu, H. Wang, S. Dong, Z.R. Tang, H. Peng, W. Zhao, C. Yin, Interpretable clinical visualization model for prediction of prognosis in osteosarcoma: a large cohort data study, Front Oncol 12 (2022) 945362.  [60] Y. Liao, Q. Liu, C. Xiao, J. Zhou, Machine learning and experimental validation to construct a metastasis-related gene signature and ceRNA network for predicting osteosarcoma prognosis, J Orthop Surg Res (1749-799X (Electronic)) (2022).  [61] H. Zhang, P. Xu, Y.A.-O. Song, Machine-Learning-Based m5C Score for the Prognosis Diagnosis of Osteosarcoma, J Oncol (1687-8450 (Print)) (2021).  [62] S.H. Kim, K.H. Shin, E.H. Park, Y.J. Cho, B.K. Park, J.S. Suh, W.I. Yang, A new relative tumor sizing method in epi-metaphyseal osteosarcoma, BMC Cancer (1471-2407 (Electronic)) (2015).  [63] J. Zhang, Z. Pan, J. Yang, X. Yan, Y. Li, J.A.-O. Lyu, A nomogram for determining the disease-specific survival in Ewing sarcoma: a population study, BMC Cancer (1471-2407 (Electronic)) (2019).  [64] Y. He, H. Liu, S. Wang, J. Zhang, A nomogram for predicting cancer-specific survival in patients with osteosarcoma as secondary malignancy, Sci Rep (2045-2322 (Electronic)) (2020).  [65] J. Wang, C. Zhanghuang, X. Tan, T. Mi, J. Liu, L. Jin, M. Li, Z. Zhang, D. He, A Nomogram for Predicting Cancer-Specific Survival of Osteosarcoma and Ewing's Sarcoma in Children: A SEER Database Analysis, Front Public Health (2296-2565 (Electronic)) (2022).  [66] L. Ye, C. Hu, C. Wang, W. Yu, F. Liu, Z. Chen, Nomogram for predicting the overall survival and cancer-specific survival of patients with extremity liposarcoma: a population-based study, BMC Cancer 20(1) (2020) 889.  [67] X.Y. Yang, X. He, Y.A.-O. Zhao, Nomogram to Predict Overall and Cancer-Specific Survival in Patients with Synovial Sarcoma in the Extremities: A Population-Based Study, Comput Intell Neurosci (1687-5273 (Electronic)) (2022).  [68] R.A.-O. Zhao, Z.A.-O. Li, Y.A.-O. Huang, C.A.-O. Xiong, C.A.-O. Zhang, H.A.-O. Liang, J.A.-O. Xu, X.A.-O. Luo, A Novel Ferroptosis-Related Gene Signature for Prognosis Prediction in Ewing Sarcoma, Anal Cell Pathol (Amst) (2210-7185 (Electronic)) (2022).  [69] C. Wu, S.A.-O.X. Gong, G.A.-O. Osterhoff, N. Schopow, A Novel Four-Gene Prognostic Signature for Prediction of Survival in Patients with Soft Tissue Sarcoma, Cancers (Basel) (2072-6694 (Print)) (2021).  [70] L. Wan, W. Zhang, Z. Liu, Z. Yang, C. Tu, Z. Li, A Novel Glutamine Metabolism-Related Gene Signature in Prognostic Prediction of Osteosarcoma, Int J Gen Med (1178-7074 (Print)) (2022).  [71] H. Ren, A.V. Bazhin, E. Pretzsch, S. Jacob, H. Yu, J. Zhu, M. Albertsmeier, L.H. Lindner, T. Knأ╧sel, J. Werner, M.K. Angele, F. Bأ╧sch, A novel immune-related gene signature predicting survival in sarcoma patients, Mol Ther Oncolytics (2372-7705 (Print)) (2021).  [72] Z. Li, H. Zheng, L. Liu, Z. Fen, H. Cao, J. Yang, J. Wei, A novel inflammatory signature for evaluating immune microenvironment status in soft tissue sarcoma, Front Oncol (2234-943X (Print)) (2022).  [73] Y. Zheng, J. Lu, Z. Shuai, Z. Wu, Y. Qian, A novel nomogram and risk classification system predicting the Ewing sarcoma: a population-based study, Scientific Reports 12(1) (2022) 8154.  [74] Q. Lin, Q. Huang, Q. Wang, W. Yan, Y. Sun, Novel Nomograms-Based Prediction Models for Patients with Primary Undifferentiated Pleomorphic Sarcomas Resections, Cancers (Basel) (2072-6694 (Print)) (2021).  [75] B. Xiao, L. Liu, Z. Chen, A. Li, Y. Xia, P. Wang, C. Xiang, Y. Zeng, H. Li, A Novel Overall Survival Prediction Signature Based on Cancer Stem Cell-Related Genes in Osteosarcoma, Front Cell Dev Biol (2296-634X (Print)) (2021).  [76] C.A.-O. Huang, Z.A.-O.X. Huang, Z.A.-O. Zhou, A Novel Prognostic Nomogram and Risk Classification System for Predicting Cancer-Specific Survival of Postoperative Fibrosarcoma Patients: A Large Cohort Retrospective Study, J Oncol (1687-8450 (Print)) (2022).  [77] W.A.-O. Chen, Y. Lin, J. Huang, Z. Yan, H. Cao, A novel risk score model based on glycolysis-related genes and a prognostic model for predicting overall survival of osteosarcoma patients, J Orthop Res (1554-527X (Electronic)) (2022).  [78] Z. Huang, Y. Wang, Y. Wu, C. Guo, W. Li, Q.A.-O. Kong, A Novel Tool to Predict the Overall Survival of High-Grade Osteosarcoma Patients after Neoadjuvant Chemotherapy: A Large Population-Based Cohort Study, J Oncol (1687-8450 (Print)) (2022).  [79] G. Wang, X. Zhang, W. Feng, J. Wang, Prediction of Prognosis and Immunotherapy of Osteosarcoma Based on Necroptosis-Related lncRNAs, Front Genet (1664-8021 (Print)) (2022).  [80] J. Jiang, H. Pan, M. Li, B. Qian, X. Lin, S. Fan, Predictive model for the 5-year survival status of osteosarcoma patients based on the SEER database and XGBoost algorithm, Scientific Reports 11(1) (2021) 5542.  [81] F.A.-O. Yang, H.A.-O. Xie, Y.A.-O.X. Wang, Prognostic Nomogram and a Risk Classification System for Predicting Overall Survival of Elderly Patients with Fibrosarcoma: A Population-Based Study, J Oncol (1687-8450 (Print)) (2021).  [82] Z. Yao, Z. Tan, J. Yang, Y. Yang, C. Wang, J. Chen, Y. Zhu, T. Wang, L. Han, L. Zhu, Z. Yang, Prognostic nomogram for predicting 5-year overall survival in Chinese patients with high-grade osteosarcoma, Sci Rep (2045-2322 (Electronic)) (2021).  [83] D. Zhang, J. Hu, Z. Liu, H. Wu, H. Cheng, C. Li, Prognostic nomogram in patients with epithelioid sarcoma: A SEER-based study, Cancer Med 12(3) (2023) 3079-3088.  [84] T. Jiang, Z. Ye, T. Shao, Y. Luo, B. Wang, Prognostic nomograms for predicting overall survival and cancer-specific survival in patients with angiosarcoma, a SEER population-based study, Sci Rep (2045-2322 (Electronic)) (2022).  [85] T.S. Goh, J.S. Lee, J. Il Kim, Y.G. Park, K. Pak, D.C. Jeong, S.O. Oh, Y.A.-O. Kim, Prognostic scoring system for osteosarcoma using network-regularized high-dimensional Cox-regression analysis and potential therapeutic targets, J Cell Physiol (1097-4652 (Electronic)) (2022).  [86] Q.A.-O. Yang, T. Chen, Z. Yao, X. Zhang, Prognostic value of pre-treatment Naples prognostic score (NPS) in patients with osteosarcoma, World J Surg Oncol (1477-7819 (Electronic)) (2020).  [87] Y. Zou, Q. Yang, Y. Wu, H. Ai, Z. Yao, C. Zhang, F. Luo, Prognosticators and Prognostic Nomograms for Leiomyosarcoma Patients With Metastasis, Front Oncol (2234-943X (Print)) (2022).  [88] Y. Shi, R. He, Z. Zhuang, J. Ren, Z. Wang, Y. Liu, J. Wu, S. Jiang, K.A.-O. Wang, A risk signature-based on metastasis-associated genes to predict survival of patients with osteosarcoma, J Cell Biochem (1097-4644 (Electronic)) (2020).  [89] B. Gao, M.D. Wang, Y. Li, F. Huang, Risk stratification system and web-based nomogram constructed for predicting the overall survival of primary osteosarcoma patients after surgical resection, Front Public Health (2296-2565 (Electronic)) (2022).  [90] Y. Chen, C. Chi, D. Chen, S. Chen, B. Yang, S. Huang, Z.A.-O. Zheng, Score for the Overall Survival Probability Scores of Fibrosarcoma Patients after Surgery: A Novel Nomogram-Based Risk Assessment System, J Oncol (1687-8450 (Print)) (2021).  [91] H. Zhan, F. Mo, M. Zhu, X. Xu, B. Zhang, H. Liu, M. Dai, A SEER-based nomogram accurately predicts prognosis in Ewingق│└s sarcoma, Scientific Reports 11(1) (2021) 22723.  [92] Z. Chen, X. Wang, G. Wang, B. Xiao, Z. Ma, H. Huo, W. Li, A seven-lncRNA signature for predicting Ewing's sarcoma, PeerJ (2167-8359 (Print)) (2021).  [93] Y. Wu, L. Xu, P. Yang, N. Lin, X. Huang, W. Pan, H. Li, P. Lin, B. Li, V. Bunpetch, C. Luo, Y. Jiang, D. Yang, M. Huang, T. Niu, Z. Ye, Survival Prediction in High-grade Osteosarcoma Using Radiomics of Diagnostic Computed Tomography, EBioMedicine (2352-3964 (Electronic)) (2018).  [94] W.A.-O. Chen, C. Zhou, Z. Yan, H. Chen, K. Lin, Z. Zheng, W. Xu, Using machine learning techniques predicts prognosis of patients with Ewing sarcoma, J Orthop Res (1554-527X (Electronic)) (2021).  [95] M.E.R. Bongers, Q. Thio, A.V. Karhade, M.L. Stor, K.A. Raskin, S.A. Lozano Calderon, T.F. DeLaney, M.L. Ferrone, J.H. Schwab, Does the SORG Algorithm Predict 5-year Survival in Patients with Chondrosarcoma? An External Validation, Clin Orthop Relat Res 477(10) (2019) 2296-2303.  [96] M.E.R. Bongers, A.V. Karhade, E. Setola, M. Gambarotti, O.Q. Groot, K.E. Erdogan, P. Picci, D.M. Donati, J.H. Schwab, E. Palmerini, How Does the Skeletal Oncology Research Group Algorithm's Prediction of 5-year Survival in Patients with Chondrosarcoma Perform on International Validation?, Clin Orthop Relat Res 478(10) (2020) 2300-2308. | | | | | | | | | | |  |
